# The adenylate cyclase gene *MaAC* is required for virulence and multi-stress tolerance of *Metarhizium acridum*

**DOI:** 10.1186/1471-2180-12-163

**Published:** 2012-08-01

**Authors:** Shuyang Liu, Guoxiong Peng, Yuxian Xia

**Affiliations:** 1 Genetic Engineering Research Center, College of Bioengineering, Chongqing University, Chongqing, 400030, P. R. China; 2 Chongqing Engineering Research Center for Fungal Insecticides, Chongqing, 400030, P. R. China; 3 Key Lab of Functional Gene and Regulation Technologies under Chongqing Municipal Education Commission, Chongqing, 400030, P. R. China

**Keywords:** Biocontrol agents, Entomopathogenic fungi, Conidia, Virulence, Environmental stress

## Abstract

**Background:**

The efficacy of entomopathogenic fungi in pest control is mainly affected by various adverse environmental factors, such as heat shock and UV-B radiation, and by responses of the host insect, such as oxidative stress, osmotic stress and fever. In this study, an adenylate cyclase gene (*MaAC*) was cloned from the locust-specific entomopathogenic fungus, *Metarhizium acridum*, which is homologous to various fungal adenylate cyclase genes. RNA silencing was adapted to analyze the role of *MaAC* in virulence and tolerance to adverse environmental and host insect factors.

**Results:**

Compared with the wild type, the vegetative growth of the RNAi mutant was decreased in PD (potato dextrose medium), Czapek-dox and PDA plates, respectively, demonstrating that *MaAC* affected vegetative growth. The cAMP levels were also reduced in PD liquid culture, and exogenous cAMP restored the growth of RNAi mutants. These findings suggested that *MaAC* is involved in cAMP synthesis. The knockdown of *MaAC* by RNAi led to a reduction in virulence after injection or topical inoculation. Furthermore, the RNAi mutant grew much slower than the wild type in the haemolymph of locust *in vitro* and *in vivo*, thus demonstrating that *MaAC* affects the virulence of *M. acridum* via fungal growth inside the host locust. A plate assay indicated that the tolerances of the *MaAC* RNAi mutant under oxidative stress, osmotic stress, heat shock and UV-B radiation was decreased compared with the wild type.

**Conclusion:**

*MaAC* is required for virulence and tolerance to oxidative stress, osmotic stress, heat shock and UV-B radiation. *MaAC* affects fungal virulence via vegetative growth inside the insect and tolerance against oxidative stress, osmotic stress and locust fever.

## Background

Fungal biocontrol agents, which are widespread and environmentally safe, have great potential in integrated pest management. However, the application of entomopathogenic fungi such as *Metarhizium acridum* in the field has been held back owing to their poor efficacy [1]. During the infection process of entomopathogenic fungi, germ tubes are produced after the fungal conidia attach to the insect cuticle, and then differentiate into swollen infection structures called appressoria. The appressoria produce penetration pegs, which penetrate the host cuticle via a combination of mechanical pressure and cuticle degrading enzymes, before piercing the surface of the host into the blood cavity. They produce a large number of hyphae through budding, thereby exhausting the nutrition of the insect host [2]. During the course of fungal infection, pathogenic fungi encounter various adverse factors from the host insect, such as antifungal substances on the cuticle [3], oxidative stress during infection [4], osmotic stress inside the host body [5], behavioral changes, such as locust fever during the early infection stage [6,7], and environmental stresses, including heat shock and UV radiation [8]. Research on the regulatory processes involved in response to adverse factors from the host and environment is essential for the commercial development and improvement of fungi as biocontrol agents.

As major regulators of virulence determinants, the signal transduction pathways of fungal pathogens have been extensively researched. In fungi and yeasts, the cAMP (adenosine 3′, 5′-cyclic monophosphate) signaling cascade has been co-opted for a multitude of cellular processes and development. cAMP regulates morphogenesis and virulence in a variety of fungi [9]. Adenylyl cyclase anchored in membrane is responsible for catalyzing the conversion of ATP to cAMP [10]. Recent studies indicate that adenylate cyclase is required for normal vegetative growth, infection structure formation and virulence in phytopathogenic fungi. The role of adenylate cyclase enzymes has been investigated in several fungal species [10-12]. *Magnaporthe oryzae* depleted of adenylate cyclase (*MAC1*) was incapable of penetrating the surface of susceptible rice leaves because it could not form appressoria [11]. In the post-harvest necrotrophic fungus *Botrytis cinerea*, the deletion of the gene encoding adenylate cyclase reduced intracellular cAMP levels, causing delayed vegetative growth, lesion development and *in planta* sporulation [12]. An adenylate cyclase (*SAC-1*) deletion mutant in *Sclerotinia sclerotiorum* exhibited aberrations in sclerotial initiation, possessed altered oxalate levels, and showed reduced virulence due to the lack of infection cushion formation [10]. Targeted disruption of the adenylate cyclase-coding gene in *Fusarium proliferatum* retarded vegetative growth, increased conidiation and delayed conidial germination [13]. Although adenylate cyclase plays various roles in a number of fungi, the function of adenylate cyclase in entomopathogenic fungi has not been explored up to date.

In this study, we cloned the full-length cDNA of adenylate cyclase from the locust-specific *M. acridum* strain, CQMa 102, designated *MaAC*. The *MaAC* transcript level of *M. acridum* was knocked-down by RNAi and the roles of *MaAC* in pathogenicity and tolerance to stresses were analyzed. Our results showed that *MaAC* contributed to vegetative growth, virulence and tolerance to various adverse host insect and environmental factors. The results demonstrated that impairment in the virulence of the *MaAC* RNAi mutant was caused by decreased vegetative growth and tolerance to adverse conditions encountered during host infection.

## Results

### Isolation and characteristics of *MaAC*

**Figure 1:**
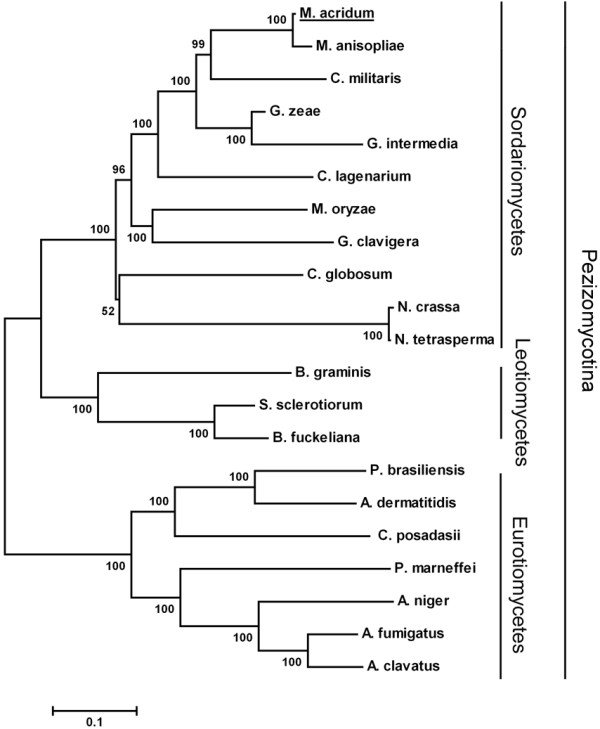
**Neighbor-joining tree inferred from *MaAC* protein sequence alignment.** Numbers on the nodes represent the results of bootstrap analyses (1,000 replicates), using the neighbor-joining method. Fungal species: *M. acridum* (JQ358775), *Metarhizium anisopliae* (EFY97222.1), *Cordyceps militaris* (EGX90508.1), *Gibberella zeae* (XP_381410.1), *Gibberella intermedia* (AAY79378.1), *Colletotrichum lagenarium* (BAD04045.1), *Magnaporthe oryzae* (AAC34139.1), *Grosmannia clavigera* (EFW99531.1), *Chaetomium globosum* (XP_001221049.1), *Neurospora crassa* (BAA00755.1), *Neurospora tetrasperma* (EGZ77248.1), *Blumeria graminis* (CAC19663.1), *Sclerotinia sclerotiorum* (ABF71879.1), *Botryotinia fuckeliana* (CAB77164.1), *Paracoccidioides brasiliensis* (AAS01025.1), *Ajellomyces dermatitidis* (XP_002624019.1), *Coccidioides posadasii* (EFW21958.1), *Penicillium marneffei* (XP_002146654.1), *Aspergillus niger* (XP_001394156.2), *Spathaspora passalidarum* (EGW29847.1), *Aspergillus fumigates* (CAC81748.1), *Aspergillus clavatus* (XP_001268121.1), *Spathaspora passalidarum* (EGW29847.1).

A 6,507 bp of cDNA encoding adenylate cyclase (*MaAC*) was isolated and sequenced (GenBank accession JQ358775). Alignment with the DNA sequence showed that the *MaAC* gene contained an open reading frame (ORF) and was interrupted by two introns located at the N terminus (955 bp to 1,291 bp) and the C terminus (6,219 bp to 6,279 bp). The complete ORF of *MaAC* encoded a predicted protein of 2,169 amino acids (aa) with a molecular mass of 542.0 kDa. An analysis using SignalP suggested that the N-terminal sequence of *MaAC* had no signal peptide. The predicted protein had a high similarity to the adenylate cyclase gene (*ACY*) of *Metarhizium anisopliae* (98% identity, EFY97222.1), the adenylate cyclase gene of *Cordyceps militaris* (98% identity, EGX90508.1), *MAC1* of *M. oryzae* (96% identity, AAC34139.1) and *SAC1* of *S. sclerotiorum* (95% identity, ABF71879.1). A fungal phylogenetic tree was established using MEGA 4.0 (Figure 1). *MaAC* was most similar to the sequence of the entomopathogenic fungus *M. anisopliae*, belonging to the *Sordariomycetes*. All species were members of the subdivision *Pezizomycotina* in the division *Ascomycota*.

### Knocked-down *MaAC* transcription by RNAi

**Figure 2:**
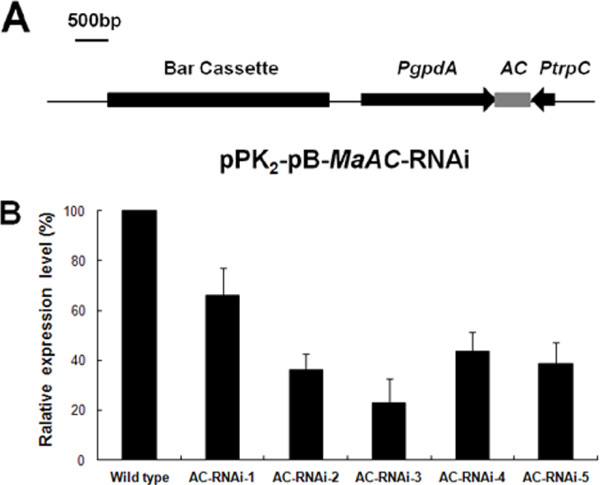
**Construction and quantitative RT-PCR analysis of the AC-RNAi mutant.A**. Maps of pPK_2_-Pb-MaAC-RNAi, the silencing vector for *MaAC*. PgpdA: promoter of gpd from *A. nidulans*; bar: herbicide resistance gene; TtrpC: terminator of trpC from *A. nidulans*; AC: partial sequence of the adenylate cyclase element gene in *M. acridum*. **B**. Relative expression of *MaAC* in the wild type (calibrated as 100%) and three RNAi strains. Error bars denote standard deviations of three trials.

We conducted an RNA interference (RNAi) strategy to study the function of *MaAC*. Phosphinothricin-resistant transformants of *M. acridum* were generated by transformation with the vector pK_2_-Pb*-*MaAC-RNAi (Figure 2A). To investigate the efficiency of RNAi, the wild type and RNAi mutants of *MaAC* were analyzed by quantitative RT-PCR. Compared to the wild type, *MaAC* transcription in the RNAi mutants was downregulated by 66.0%, 43.5%, 23.1%, 36.2% and 38.8%, respectively (Figure 2B). These results demonstrated that the transcription of *MaAC* was efficiently knocked down.

### *MaAC* affects growth *in vitro*

**Figure 3:**
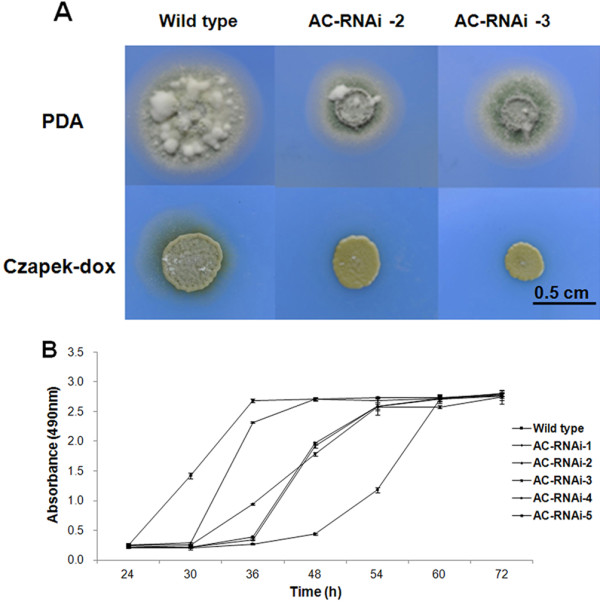
**Effect of *MaAC* on vegetative growth in the wild type and AC-RNAi mutants.A**. The colonies were cultured on PDA and Czapek-dox medium for 10 d. Scale bar: 0.5 cm. **B**. The OD_490_ after a 3-h incubation of the wild type and AC-RNAi mutant cultured for 72 h mixed with CellTiter 96® AQueous One Solution Reagent in PD liquid culture. Error bars denote the standard deviations from three trials.

The phenotypes of the *MaAC* RNAi mutants *in vitro* were analyzed on PDA and Czapek-dox medium (Figure 3A). A variety of morphological abnormalities were observed in the *MaAC* RNAi mutants. On PDA, the growth of the *MaAC* RNAi mutants was reduced, mycelium formation was delayed, and the colonies of RNAi mutants were smaller compared to the wild type. On Czapek-dox medium, the conidiation of the *MaAC* RNAi mutants was also delayed, and the colonies of RNAi mutants were lighter in comparison to the wild type. The AC-RNAi-3 mutant had the most significant difference compared to the wild type, and was used as the *MaAC* RNAi mutant in the following experiments.

Vegetative growth *in vitro* was further quantified by assaying the living cells in PD liquid culture by CellTiter 96® AQueous One Solution Assay (Figure 3B). In contrast to the wild type, the growth rate of the AC-RNAi-1 mutant was similar to the wild type, while the other four RNA mutants grew conspicuously slowly (p <0.01). These results indicated that *MaAC* affects growth *in vitro*. The correlation coefficient of the relative expression rate and the growth rate was 0.94, which was highly significant (p <0.01). These result showed that the growth rate is related to the relative expression rate of *MaAC*.

### *MaAC* regulates intracellular cAMP levels in *M. acridum*

**Figure 4:**
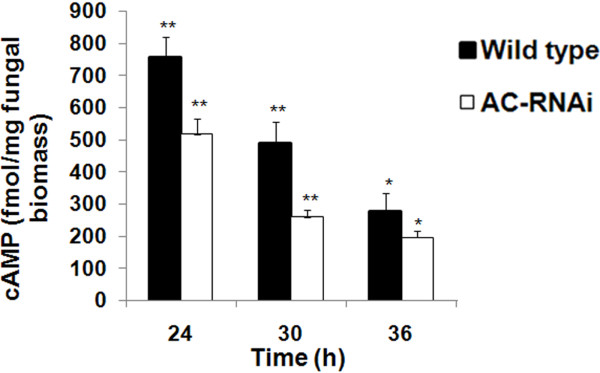
**cAMP levels in the AC-RNAi mutant and wild type strains.** Freshly harvested conidia (10^7^ cells per mL) of AC-RNAi mutant and wild types were cultured in PD liquid culture, and harvested at 24, 30 and 36 h for estimation of the cAMP levels. Standard deviation bars denote averages from three independent experiments. *: significant difference, p <0.05; **: significant difference, p <0.01.

**Figure 5:**
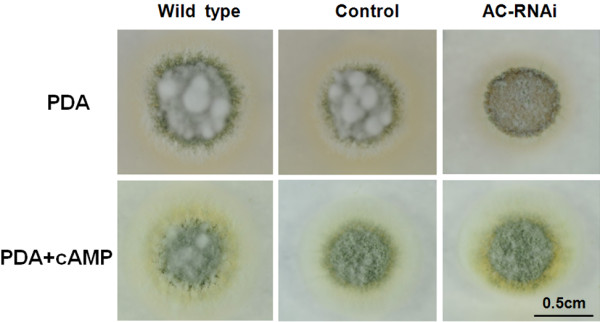
**Exogenous addition of 8-Br-cAMP to the AC-RNAi mutant results in increased growth rates.** The morphology of the wild type, knockdown control and AC-RNAi mutant colonies grown in the presence of 8-Br-cAMP (5 mM) were inoculated on PDA medium. These cultures were grown for 5 d prior to documentation. Scale bar: 0.5 cm.

As shown in this study, the fungal growth of the *MaAC* RNAi mutant of *M. acridum* was significantly slower *in vitro* than that of the wild type. In order to assess whether the growth defect of the RNAi mutant was due to reduced levels of cAMP, we quantified and compared the steady-state levels of cAMP in PD liquid culture. The cAMP level was significantly reduced in the AC-RNAi-3 mutant compared to the wild type (Figure 4A) and the cAMP concentration of the *MaAC* RNAi mutant (259.4 fMol/mg) was approximately two-fold less than that of the wild type (486.8 fMol/mg) after being cultured for 30 h (p <0.01). This demonstrated that *MaAC* was involved in cAMP production during the vegetative growth of *M. acridum*. This was further confirmed by the exogenous addition of cAMP (8-Br-cAMP) to the RNAi mutant. As shown in Figure 5, the RNAi mutant grown in the presence of 8-Br-cAMP showed a great increase in aerial hyphal growth. Thus, exogenous cAMP could restore the growth of the RNAi mutant, which suggested that *MaAC* was involved in cAMP synthesis.

### *MaAC* is required for *in vivo* virulence and growth

**Figure 6:**
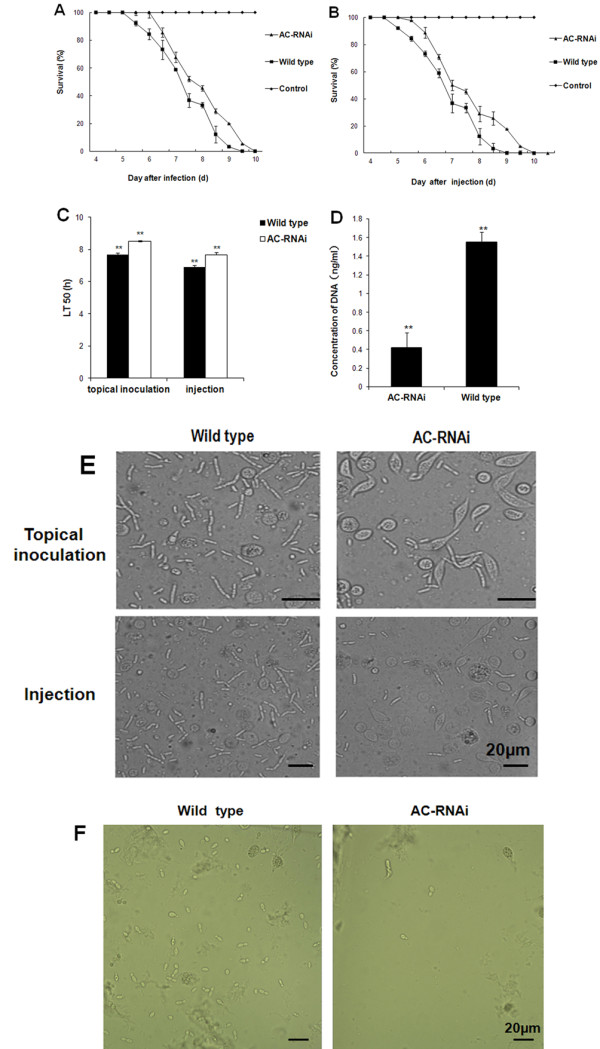
**The virulence and fungal growth in the haemolymph of locust *in vivo* and *in vitro* .A**. Topical application with 5 μL suspensions of 1 × 10^7^ conidia/mL of wild type and RNAi mutant (control insects were inoculated with 5 μL cottonseed oil). **B**. Survival of the locusts by injection with 5 μL suspensions of 2 × 10^6^ conidia/mL (control insects were injected with 5 μL sterile water). **C**. Lethal time for 50% mortality (LT_50_) values of *Locusta migratoria* treated with the wild type or AC-RNAi mutant. Error bars denote standard deviations obtained from five trials. **D**. DNA concentration of AC-RNAi and wild type in the hemolymph of locusts 48 h after injection. **E**. Photomicroscopy of the development of conidiation patterns of *M. acridum* in the hemolymph of locusts. After 4 d of infection on the pronotum, the conidiation of the RNAi mutant strain grew slower than the wild type strain. The conidiation of the RNAi mutant strain grew also slower than the wild type strain 3 d after injection into abdominal segments. **F**. Photomicroscopy of the development of conidiation patterns of *M. acridum* in the hemolymph of locusts *in vitro*. After they were cultured for 24 h, the conidiation of the RNAi mutant strain grew slower than the wild type strain. Scale bar: 20 μm. Error bars are standard deviations of five trials. *: significant difference, p <0.05, **: significant difference, p <0.01.

Differences in virulence and invasive growth inside insects were also compared between the wild type and RNAi mutant. Figure 6A shows that, 5 days post-inoculation on the pronotum, locusts infected by the wild type fungus began to die, while those infected by the RNAi mutant died 1 day later. Figure 6B shows that when the insects were inoculated by the injection of conidia into abdominal segments, the locusts began to die 4 days after injection of the wild type, and again the insects treated with the conidia of RNAi mutant died 1 day later. Accordingly, the lethal time value for 50% mortality (LT_50_) by topical inoculation and injection of the RNAi mutant was significantly higher than that of the wild type (p <0.05) (Figure 6C), which indicated that *MaAC* is required for *M. acridum* virulence.

To confirm the effect of *MaAC* on virulence, fungal growth *in vivo* was observed by photomicroscopy and quantified by real-time PCR. The *M. acridum* mutant grew significantly more slowly than the wild type (Figure 6E), which was further confirmed by a quantitative assay. Here, the fungal DNA of the wild type was conspicuously higher (~4 times) than that of the RNAi mutant (Figure 6D). Fungal growth cultured in the haemolymph of the locusta *in vitro* was also observed by photomicroscopy, which showed that the RNAi mutant grew evidently more slowly than the wild type (Figure 6F). Taken together, these results demonstrate that *MaAC* affects fungal growth both *in vivo* and *in vitro*.

### *MaAC* is involved in the tolerance of *M. acridum* to oxidative stress and osmotic stress

**Figure 7:**
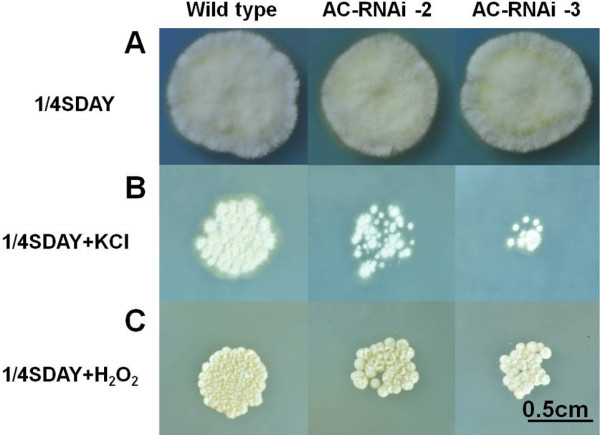
**Growth characterization of AC-RNAi mutants and wild type *M. acridum* with oxidative or osmotic stresses.A**. Colonies of wild type and AC-RNAi mutants were cultured on 1/4SDAY medium for 10 d. **B**. Colonies of wild type and AC-RNAi mutants were cultured on 1/4SDAY + KCl (1 M) medium for 10 d. **C**. Colonies of wild type and RNAi strains were cultured on 1/4SDAY + H_2_O_2_ (6 mM) medium for 10 d. Scale bar: 0.5 cm.

In order to clarify the mechanisms by which *MaAC* affect the virulence and growth *in vivo*, the osmosensitivity and H_2_O_2_ tolerance of conidia were analyzed. Firstly, 1/4 SDAY was chosen as a base medium, on which these strains grew with no difference 10 d post-inoculation (Figure 7A). However, RNAi mutants were more sensitive to osmotic stress, and the RNAi mutants colonies were sparse in contrast to the dense ones of the wild type on 1/4 SDAY + KCl (1 M) (Figure 7B). The effect of externally applied H_2_O_2_ on the wild type and RNAi mutants was also tested (Figure 7C). The most striking differences between the response of the wild type and RNAi mutants was observed in 1/4 SDAY containing 6 mM H_2_O_2_, where the colonies of the RNAi mutants were sparser than the wild type colonies. These results indicated that *MaAC* is involved in the tolerance of *M. acridum* to both oxidative and osmotic stresses.

### *MaAC* affects the tolerance to heat and UV light

**Figure 8:**
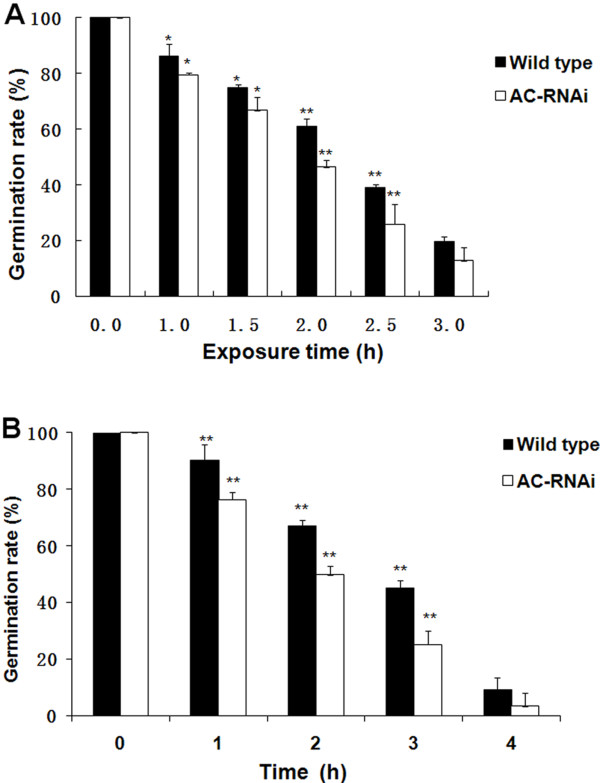
**Germination rate of the *M. acridum* wild type and AC-RNAi mutant with wet-heat and UV-B treatments.A**. Germination rate were tested after wet-heat exposure to temperature of 45°C for 0, 1.0, 1.5 2.0, 2.5 and 3.0 h. **B**. Germination rate after UV-radiation exposure for 0, 1, 2, 3 and 4 h. Standard deviation bars denote standard deviations for three independent experiments. *: significant difference, p <0.05; **: significant difference, p <0.01.

The tolerance levels of conidia to heat and UV light were analyzed to clarify the function of *MaAC*. After wet-heat exposure at 45°C, the germination rate of conidia declined with increasing exposure times, and the conidia germination rates of the wild type strain and mutants appeared to be significantly reduced for each successive 30-min interval (Figure 8A). However, the response to tolerance was obviously different for the wild type strain and RNAi mutant. The conidia germination rate of the wild type strain was higher than that of the mutant. In particular, there was a significant difference at 2 h and 2.5 h (p <0.01). Similar results were observed with the UV-B tolerance test (Figure 8B). Exposure to UV-B for 1–3 h caused a significant difference in the germination rate of conidia between the wild type and RNAi mutant (p <0.01). These result indicated that the RNAi mutant was more sensitive to UV-B treatment than the wild type. Therefore, *MaAC* appears to affect the tolerance of *M. acridum* to heat and UV.

## Discussion

Adenylate cyclase regulates a variety of physiological processes in phytopathogenic fungi, including conidiation, conidial germination, vegetative growth, appressoria formation and virulence. In this study, an adenylate cyclase gene, *MaAC*, was identified in a locust-specific entomopathogenic fungus, *M. acridum*. Bioinformatic analysis showed that the cloned *MaAC* had significant similarity to its homolog from *M. oryzae* and to many other fungal adenylate cyclase genes; the highest degree of similarity was found with the adenylate cyclase of *M. anisopliae* (98% identity). The cAMP level of the *MaAC* RNAi mutant was significantly reduced, and the exogenous addition of cAMP could restore the growth of the RNAi mutant, thus confirming that the *MaAC* gene encodes adenylate cyclase in *M. acridum*. These results were similar to previous studies on other fungi [10,12,14]. Following the deletion of the entire *SAC1* coding sequence of *S. sclerotiorum*[10], cAMP underwent a four-fold reduction in the *SAC1* deletion strain compared to the wild type. In *BAC1*- and *UAC1*-defective mutants, intracellular cAMP was detected, which contrasted with the wild type [13,15].

In this report, the downregulation of *MaAC* led to inhibited growth on *in vitro* media, including PDA and Czapek-dox medium. In PD liquid culture, it caused similar effects to previously described adenylate cyclase mutants, such as the *SAC1* mutant in *S. sclerotiorum*[10] and the *BAC1* mutant in *B. cinerea*[12]. Furthermore, *MaAC* is also involved in the growth of *M. acridum* inside locusts. The virulence of the *MaAC* mutant was also significantly reduced, thus indicating that *MaAC* is required for *M. acridum* virulence. This finding is consistent with the role of adenylate cyclase in the virulence of other fungi, including *M. oryzae*[11], *B. cinerea*[12] and *U. maydis*[15].

Previous research has demonstrated that the tolerance of fungi to stresses such as high temperature [13], UV-B radiation [8,16], oxidative [13] and osmotic stress [4,5,17] is a factor that limits their widespread use. The elevated thermo- and H_2_O_2_-tolerance of the *ΔFpacy1* mutants indicated that the adenylate cyclase may have negative regulatory roles on the stress response mechanisms of fungal cells [13]. However, the tolerance of the RNAi mutant to the osmotic-, H_2_O_2_-, UV-B and thermal stress was reduced in this study, thus indicating that *MaAC* may affect the tolerance to multiple stresses through similar regulatory mechanisms in fungal cells.

*MaAC* affects virulence via controlling the rate of vegetative growth inside the host locust and the tolerance of the fungus to oxidative stress, osmotic stress and locust fever (immune response). The ΔLT_50_ values of the AC-RNAi mutant and the wild type after topical inoculation and injection were similar (p >0.05), but the germination and appressorium formation of the AC-RNAi mutant was not affected (Table 1). The fungal growth of the AC-RNAi mutant *in vivo* and *in vitro* was slower compared to the wild type, thus resulting in a reduction of virulence as a result of the slow growth of the AC-RNAi mutant in the host body. The effect of adenylate cyclase on virulence is mediated by different mechanisms in different pathogenic fungi. For example, the virulence effect of the *MAC1* mutation is due to the inability of the fungus to produce appressoria [11], while the effect of the *BAC1* mutation on virulence is due to the absence of sporulation in plants [12]. A fungal pathogen would encounter oxidative stress during infection or osmotic stress inside the host body [4,5], and locust fever (immune response) during the early stage of infection [6,7]. Therefore, the effect of *MaAC* on stress tolerance in the host insect contributes significantly to the virulence of *M. acridum*.

**Table 1:** Germination and appressoria formation on locust wings

	**Germination rate**^**a**^**(%)**		**Appressorium formation rate**^**b**^**(%)**	
	**Wild type**	**AC-RNAi-3**	**Wild type**	**AC-RNAi-3**
	**Wild type**	**AC-RNAi-3**	**Wild type**	**AC-RNAi-3**
14h	33.3 ± 4.7	25.0 ± 5.6	0	0
18h	55.7 ± 4.0	40.3 ± 1.5	0	0
24h	80.6 ± 6.1*	66.3 ± 6.5*	53.7 ± 5	48.3 ± 3
28h	99.3 ± 1.7	98.0 ± 2.9	79.6 ± 5	77.6 ± 4

a. The germination rate of the wild type and AC-RNAi-3 cultivated on locust wings for 28h.

b. The appressorium formation rate of the wild type and AC-RNAi-3 cultivated on locust wings for 28h.

*: Significant difference at a value of p <0.05.

## Conclusions

An adenylate cyclase encoding gene (*MaAC*) was cloned from the locust-specific entomopathogenic fungus, *M. acridum*. *MaAC* affects virulence and fungal growth inside the insect, and is required for its tolerance to oxidative stress, osmotic stress, heat shock and UV-B radiation. *MaAC* affects fungal virulence via vegetative growth and tolerance to oxidative stress, osmotic stress and locust fever.

## Methods

### Strain and culture conditions

*M. acridum* strain CQMa102 was isolated from infected yellow-spined bamboo locusts **(***Ceracris kiangsu* Tsai**)** and was used to derive all strains in this study [18]. The conidia were collected after the fungus was cultured on 1/4 strength Sabouraud’s dextrose agar yeast medium (1/4 SDAY; 1% dextrose, 0.25% mycological peptone, 2% agar and 0.5% yeast extract, w/v) at 28°C for 15 d. The medium used for growing mycelia was PD (potato dextrose medium) liquid culture. Czapek-dox medium (3% saccharose, 0.2% NaNO_3_, 0.1% K_2_HPO_4_, 0.05% KCl, 0.05% MgSO_4_, 0.001% FeSO_4_) and potato medium (PDA, 20% potato, 2% sucrose, 2% agar) were used for colony phenotype testing.

### Gene cloning, phylogenetic analysis and construction of the *MaAC* RNAi vector

Genomic DNA of *M. acidum* was extracted as previously described [19]. The genomic DNA sequence was acquired by PCR with the primer pairs (5′-TTCCACGCCAAACCTCAA -3′) and MaAC-R (5′-AGCCAAGTTGTTTCGGTA -3′) from the whole genomic sequence of *M. acidum*[20]. A full-length cDNA clone of *MaAC* was amplified using Pyrobest DNA polymerase (TaKaRa, Japan) from a cDNA library of *M. acridum* established in our laboratory [21] with gene-specific primers MaAC-F (5′- TTCCACGCCAAACCTCAA -3′) and MaAC-R (5′- AGCCAAGTTGTTTCGGTA -3′). The resulting PCR product was subcloned into the pMD19-T vector, and transformed into *E. coli XL-Blue* for determination by GenScript (Nanjing, China). To study the function of *MaAC*, an RNA interference (RNAi) vector was constructed. The partial sequence of *MaAC* (500 bp) was amplified by MaAC-F (5′- GCGATACACGCCACAAGGACAAAGA-3′) and MaAC-R (5′-CCCAAGCTTACTACCAATCTCATCCACCTC-3′) from *M. acridum MaAC* cDNA. The resulting PCR product was cloned into pMD19-T (Takara, China) to form pMD19-MaAC. A fragment of *MaAC* was recovered from pMD19-MaAC by digestion with *Eco*RI and *Eco*RV and inserted into the vector pDPB [22]. The fragment PgpdA-MaAC-PtrpC from pDPB was inserted at the site between *Hind*III and *Xba*I of pPK2-pB [23,24] to form pPK_2_-pB-MaAC-RNAi. Transformation of *M. acridum* was mediated by *Agrobacterium tumefaciens* according to the procedure described previously [25]. Transformants were screened on Czapek-dox medium containing 80 μg/mL phosphinothricin (PPT) and incubated at 27°C for 8 d. Transformants were confirmed by PCR amplification of the RNAi cassette.

### Real-time quantitative reverse transcript (qRT-PCR) analysis

To confirm the expression levels of *MaAC*, the wild type and MaAC-RNAi transformants were grown in PD liquid culture for 2 d and the mycelia were collected and washed with sterile water. Total RNA was isolated using the SV Total RNA Isolation System (Promega, USA). The synthesis of cDNA and real-time RT-PCR were performed using the method described by Leng *et al*. [26]. Primers of MaAC-F (5′- GGACGAAGGACTTGACAGACC-3′) and MaAC-R (5′-CACAGCATCTCCAGACGAGG-3′) were used to detect *MaAC* expression levels.

### Determination of fungal growth

To characterize the role of *MaAC* in vegetative growth, the growth rate of the wild type and the RNAi mutants were analyzed using CellTiter 96® AQueous One Solution Assay (Promega, USA). In this study, the wild type or RNAi mutants were inoculated in PD liquid culture for 24, 30, 36, 48, 54, 60 and 72 h, respectively. CellTiter 96® AQueous One Solution Reagent (20 μL) and 100 μL culture fluid were directly added to the culture wells, the mixture was incubated for 2 h at 37°C, and then the absorbance was recorded at 490 nm with a 96-well plate reader.

### cAMP assay

The *MaAC* mutant and the wild type were cultured in PD liquid culture for 36 h. After harvesting, 20 mg mycelia were collected and washed three times with sterile water, followed by treatment with 2 mL 0.01 M PBS. Samples were then lyophilized and dissolved in the mixture. They were centrifuged at 4,000 rpm for 20 min and the supernatant was collected for the test. The cAMP levels were measured with cAMP Enzyme Immunoassay Kit (Sigma, USA), according to the manufacturer’s instructions. In total, each assay was repeated three times independently with three biological replicates for every strain.

To test whether exogenous cAMP could restore the growth of RNAi mutant, the cAMP analog, 8-Br-cAMP (Sigma, USA) was added to PDA at a final concentration of 5 mM. 8-Br-cAMP (a membrane permeable variant of cAMP) has been extensively used in various studies to artificially cause the enhancement of endogenous cAMP levels [27-29].

### Biomass assay and fungal growth in the haemolymph of locust *in vivo* and *in vitro*

The virulence of the RNAi mutant and the wild type was tested by topical inoculation and injection into *Locusta migratoria* adults reared under crowded conditions as previously described by He et al. [30]. The *Locusta migratoria* used were all male adult 3 days post-molt. Wild type and RNAi mutants were incubated at 28°C on 1/4 SDAY plates for 15 d. Aliquots of 5 μL solution of 10^7^ conidia/mL of either wild type *M. acridum* or RNAi mutant in cottonseed oil were inoculated on the pronotum. Aliquots of 5 μL suspensions (2 × 10^6^ conidia/mL) in sterile water were injected into the hemocoel. Both experiments were repeated five times with 30 insects per replicate. Mortality was recorded every 12 h after topical inoculation and injection. Mortality was then recorded daily, and lethal time values for 50% mortality (LT_50_) values were used to estimate the infectivity of *M. acridum* by DPS software [31]. The growth of *M. acridum* in the host locust was quantified by the detection of fungal rDNA in the infected locust using real-time PCR [32]. After the extraction of *M. acridum* DNA and fungal DNA from the infected locust, fungal DNA was detected by an Icycler iQ Quantitative PCR was performed using specific primers of *M. acridum*: CQMaP-F1: 5′-TGGCATCTTCTGAGTGGTG-3′and CQMaP-R1: 5′-CCCGTTGCGAGTGAGTTA- 3′. To test the fungal growth in the haemolymph of locust *in vitro,* 50 μL of a conidial suspension (1 × 10^7^ conidia/mL) was inoculated into 950 μL of locust haemolymph, and the growth of the wild type and mutant was detected 24 h post inoculation.

### Germination and appressoria formation against insect cuticles

The percentage of germination of wild type and RNAi mutant were measured as described by Liu *et al.*[18]. The appressorium formation rates were determined from 300 conidia after an 18 h induction on locust hind wings according to He and Xia [33]. The assay was replicated at least three times.

### Oxidative stress, osmotic stress, heat shock and UV-B treatment test

Growth characterization of the wild type and RNAi mutants were carried out on 1/4 SDAY supplemented with H_2_O_2_ (6 mM) or KCl (1 M). Samples of conidial suspensions (2 μL; 5 × 10^5^ conidia/mL) were spotted on each Petri dish and the plates were incubated at 28°C for 10 d. The tolerance to wet-heat shock and UV-B treatment of the RNAi mutant and wild type was analyzed as described by Liu et al. [22].

## Abbreviations

UV-B, Ultraviolet-B radiation; RNAi, RNA interference; 1/4SDAY, 1/4 strength Sabouraud’s dextrose agar medium; PD, Potato dextrose medium; PDA, Potato dextrose agar medium; PBS, Phosphate Buffered Saline; LT50, Lethal time of 50%; DPS, Data Processing Standards.

## Competing interests

The authors declare that they have no competing interests.

## Authors’ contributions

YX designed the research; SL and GP performed the experiments; SL, GP and YX wrote the manuscript. All authors read and approved the final version of the manuscript.
